# Therapeutics

**Published:** 1824-06-01

**Authors:** 


### Therapœia.

Pharmaca nulla valent, nisi quæ sint commoda causis.

---

1. *Antimony in the Phlegmasiæ.* A mystery and doubt still hang over the Italian mode of treating the phlegmasiæ by large doses of the tartrite of antimony. Dr. Fontaneilles has recently published his testimony respecting this litigated question, in the Archives Generales for February last, from which we shall abridge some of the particulars. We need hardly remark that the employment of tartar emetic is no new practice in the phlegmasiæ—but it was only considered as an auxiliary to blood-letting and other evacuations. The peculiarities of the Italian practice, and especially that of Russori, consist, 1$^{mo.}$ in treating pneumonia, from beginning to end, with this medicine—2$^{do.}$ in making it the principal, or almost the sole remedy—3$^{tio.}$ to thus reduce, or rather discard blood-letting.—4$^{to.}$ to exhibit the medicine in doses that would appear frightful to former practitioners—that is, to the amount of a scruple, or even some drachms in the 24 hours, and that without producing either vomiting or violent purgation.

The first accounts of this practice staggered the beliefs of physicians, who insisted that some fallacy must have crept into the reports. It was said that the medicine could not have been of its proper strength, or that the patients could not have swallowed the doses that were prescribed. Both these doubts have been cleared away, if we can credit the assertions of Dr. Fontaneilles (Physician of the Military Hospital of Milan) who has given the medicine to be analysed and experimented on by those who were competent to the task of both. Many physicians scrutinized the exhibition of the remedy in the doses described—without being able to detect any fallacy in the practice.

Our author observes that this capability of sustaining such large doses of emetic tartar depends entirely on a certain morbid state of the system—for when this morbid condition is removed, the power of taking the remedy in large doses with impunity or advantage, immediately ceases also. This, he insists, is the true and only solution of the enigma; and this indeed is most indubitably the cause, as we have hinted in a former number of this journal. This morbid condition, or diathesis, which bears the reception of such large doses of the antimony, varies in different cases of peripneumony (for it is of peripneumony which our author treats in this paper) and in different stages of the same case. Thus, this diathesis is less prominent in the beginning and in the decline of the inflammation—and most powerful at the acmé of the disease. The doses of the medicine must be regulated according to these epochs, otherwise their common effects will be produced. In general our author commences with not less than twelve grains in the day, and the same quantity in the course of the night. But when he finds that the pneumonia has made progress, he gives from a scruple to half a drachm in each of the above periods, gradually augmenting the quantity, according to the violence of the disease. Sometimes, but merely as exceptions to general rules, the dose of the medicine exceeds the ratio of the diathesis, and then vomiting is the result. At other times, the violence of the symptoms, as the cough, pain, dyspnœa, &c. will abate, while the force of the diathesis remains undiminished—as known by the capability of bearing the large doses still continuing. We must not therefore diminish the dose with the diminution of the symptoms—*unless we find the power of bearing the medicine also diminish.* Again, some of the troublesome symptoms may persist, notwithstanding that the diathesis is declining. "Thus the breathing may become more short or laborious, or delirium and tendency to coma may supervene—and if, in these cases, we find a diminished aptitude in the constitution to bear the large doses of antimony, we may conclude that organic changes are taking place, which will set at nought the resources of our art."

The cessation of the diathesis is soon proved by the patient not being able to bear the smallest quantity of the tartar emetic, without loathing and disgust, although previously swallowing large quantities without the smallest inconvenience, but, on the contrary, benefit. Although our author looks upon emetic tartar as a direct sedative, and in this respect similar to digitalis, yet he observes that its exhibition, in full doses, is not attended with the same bad effects as sometimes attend the latter medicine. He has never seen the pulse reduced below 50 in the minute by antimony, and but rarely any irregularity in the action of the heart.

Our author has been asked why he does not abandon blood-letting altogether, since he has found so effectual a remedy for pneumonia in emetic tartar? His answer to this question is, we think, a very wise one. "In many cases, says he, the pneumonia runs its course so rapidly that the structure of the lungs is menaced with destruction, which circumstance induces me to put in force, *simultaneously*, different means of reducing the phlogistic diathesis." On the other hand, he deprecates the substraction of so many pounds of blood in this disease, when venesection may be so powerfully aided and greatly abridged by the use of antimony. He properly impresses it on practitioners that blood-letting is never to be dispensed with in this class of maladies, especially when they are severe, since the effects of this remedy are prompt and certain, where time is so precious;—whereas, it requires a certain period to elapse before the antimony produces its operation in checking the inflammatory process.

Upon the whole, we think some advantage may be obtained by attending to the experience of the Italian physicians on this point of practice. The auxiliary aid of antimony has been too much despised by some modern practitioners in this country, in the treatment of internal inflammations, seduced by the powerful remedy which they possess in venesection, but which is not without its inconveniences, not to say dangers, when carried to a great extent.

---

2. *Dissolution of Calculi by Galvanism.* As long as calculus in the bladder is a painful disease, and lithotomy a formidable operation, the ingenuity of man will be at work to find out the means of mitigating the one and superseding the other. The attempt of Messrs. Prevost and Dumas to solve the stone, while in the living bladder, by the action of the galvanic fluid, appears to us to be not a very promising experiment, as far as the human frame is concerned. We shall, however, give some account of the experiments of these very ingenious and zealous physiologists.

A fusible human calculus was submitted to the action of a galvanic pile, of 120 plates, for the space of twelve hours. The calculus was placed in a vessel of pure water. The platinum wires, which served for poles, touched the opposite sides of the calculus. During the action of the battery the bases and phosphoric acid came to their respective poles, recombined, and the salt thus formed, precipitated to the bottom of the vessel in the form of a fine powder. Before the experiment, the calculus weighed 92 grains—after it 80 grains. The galvanism was continued till the end of sixteen hours, when the calculus presented only a friable mass, which was easily reduced into small crystalline bodies by the slightest pressure.

The result of this experiment naturally led to another, still more curious. A calculus was fixed on a sound, between two platinum conductors, and the apparatus introduced into the bladder of a dog, by means of an incision in the urethra under the pubic arch. The bladder was injected with warm water, which was prevented from returning. A battery of 135 plates was then brought to act on the wires. The animal was, at first, restless, but soon became quiet, and supported the action of the pile during an hour. The apparatus was cautiously withdrawn, and the calculus showed evident marks of decomposition. The experiment was then renewed and continued six days, one hour in the morning and one in the evening. By this time the calculus had become so friable that the experiment was obliged to be discontinued. It had lost in weight proportionately to the other calculus. The animal was now killed in order to examine the state of the bladder. Nothing remarkable could be discovered in this organ.

Our authors hope that means will be found out to apply the galvanic action to those calculi in the human bladder which are composed of saline principles—but they are aware that it would be quite useless to hope for any beneficial result in cases where the calculus consists wholly or principally of uric acid.

---

3. *Sulphate of Quinine in Infantile Erysipelas.* In the 3d number (New Series) of the Repository, Mr. Miles has stated a severe case of erysipelas phlegmonodes in a child, which was treated with sulphate of quinine. The child was only three weeks old, when a blush of inflammation was observed on the right side of the neck which extended by the following morning across to the opposite side, and downwards over a portion of the breast, assuming the phlegmonoid character. In a few days the whole surface was affected, with some sloughy appearances, especially about the trunk, and considerable œdema in the extremities. From extreme irritation the infant became convulsed. The subcarbonate of ammonia was tried, with extract of bark, for four days, but it had no control over the disease, and the stomach began to reject it. The sulphate of quinine was substituted, half a grain four times a day. This, Mr. Miles thinks, "supported the system in a surprising degree, and prevented, in all probability, the supervention of extensive sphacelation." Suppuration of the cellular membrane about the shoulders of the child afterwards took place, but the infant ultimately recovered.

---

4. *Porrigo Galeata, or Scald Head.* Banyer's ointment is strongly recommended for the cure of this disagreeable complaint by several practitioners, and lately by Mr. Sprague. The following is its composition.

℞. Submur. hydrarg. . . . ʒij.

Aluminis exsiccat. . .

Plumbi subcarb. . . āā ℥ss.

Terebinth. venet. . . . ʒvi.

Cerat. Cetacei . . . . ℥iss.

Misce fiat unguentum.

Dr. Chapman employs the following application:—

℞. Sulphuris sublimat. . .
Unguenti picis liq. . āā ℥iss.
Saponis mollis . . . .
Ammoniæ muriatis . āā ℥ss.
Misce fiat unguentum.

*Vide Sprague in Med. Repos.*

---

5. *Colchicum.* Dr. Kinglake has lately favoured the public with some speculations "on the medicinal properties of colchicum," in which we observe much that appears to us obscure, and something not entirely free from error. He seems to view the agency of colchicum on the human frame as quite similar to that of digitalis, hyoscyamus, cicuta, belladonna, and "other kindred articles"—that is, he considers it merely as a *narcotic*, "impairing the natural tone and *healthy* condition of nervous sensibility," without removing the *cause* of pain. Now, in the first place, we cannot bring ourselves to view colchicum as a mere narcotic, seeing that it does not (at least very rarely does) remove pain at all at the beginning of its exhibition, but only after it has produced some evacuation from the system, by the skin, kidneys, or bowels. Is this the case with the other narcotics abovementioned? Surely not. *They* will indeed lessen the sensibility of the nerves to pain, without removing the cause, which generally acts with greater force after the operation of the anodyne. But let us bring the agency of the medicine to a familiar and practical test. A man has gout in his foot, which is swelled, red, and painful. He takes a few doses of colchicum, and the pain, together with the inflammation, vanishes, generally after some increase of the natural secretions or excretions. Is this a mere "state of negative sensibility" rather "than a positive removal of morbid pain?" Will any unbiassed observer deny that, in the case abovementioned, which is an every day occurrence, the cause (inflammation) of the pain is removed along with the pain itself? The comparison, therefore, of colchicum with the common narcotics, appears most forced, unnatural, and erroneous. Indeed we do not see the propriety of ranking digitalis and belladonna together, as if they were both mere narcotics. Surely it is a perversion of observation to view digitalis simply as a narcotic. If Dr. Kinglake had a pain in his bowels would he take a dose of digitalis on the same principle and with the same expectation as he would a grain of opium or five grains of hyoscyamus? We rather think that the Doctor's lucubrations on paper would harmonize badly with his practice at the bed-side in such a case. The fact is, that every individual medicine has a peculiar or specific effect on the animal economy—and that no two, even of the purest narcotics, have a precisely similar physiological action. Will any man of practical observation say that the action of opium and henbane is the same? Both, it is true, will ease pain; but they have various other properties entirely opposed to each other. As for digitalis, we really do not know a single property which it has in common with the narcotics among which it is ranked by Dr. K.—when taken in proper doses. An ounce of tincture of digitalis will kill a man, and so will an ounce of laudanum; but this is not, we would imagine, the way to classify medicines. If so, sulphuric acid, arsenic, and almost every potent medicine may be ranked as *narcotics*, if taken in doses to produce the final sleep.

In one view of the following position, we believe, no man will venture to disagree with Dr. Kinglake. "Painful sensation is commonly rather an *effect* than the cause of disease." Sensation of every kind, painful or pleasurable, must be the *effect* of some impression on the nervous structure; but, after being an *effect*, it may itself become a *cause*.

of disease. Pain may and does derange the functions of circulation, digestion, and secretion—in short, what function does it not disturb, when excessive? Dr. Kinglake appears to view the narcotic action of colchicum as consisting in its power "of reducing inordinate vascular action, by which the sensorial principle of life is painfully agitated." If this be its modus operandi in relieving pain, then Dr. Kinglake's objection to it must apply also to blood-letting, purging, nay, even to his own favourite remedy of cold water.

Be it remembered, that we are not here advocating the treatment of gout by colchicum; but only making some observations on Dr. Kinglake's physiological principles, as explanatory of the modus agendi of the medicine. We have long been of opinion that the *causes* of gout are deeply laid in the constitution, and that the *phenomena* of gout in the extremities are no other than salutary operations to remove or obviate these causes. These salutary operations of Nature, like many other of her salutary operations, require to be controlled occasionally. But when they are suddenly and often interfered with, whether by colchicum, cold, or any other powerful means, the effects will be, sooner or later, injurious, if not fatal. In the following sentiments we agree with our author. "Gouty and rheumatic excitement, as well as every other description of painful disease, should be brought within safe and tolerably quiet bounds by vascular depletion, diminished temperature, and a lowering unirritating diet, rather than by an agency such as colchicum affords." For some other good remarks on the exhibition of the medicine we refer to the journal in which the paper is published.—*London Med. and Phys. Journal, March* 1824.

---

6. *Muriate of Lime.* Dr. Carter, in his last valuable Hospital Report, recommends a solution of muriate of lime in cases of intestinal worms. In cases of ascarides and lumbrici, after the bowels are well cleared out, the medicine appears to Dr. Carter to be an excellent remedy. In two or three instances he has known it expel worms when purgatives had failed to do so.

---

7. *Sulphate of Quinine in Cephalalgia.* Mr. Bushell has related a case, in the March number of the London Medical and Physical Journal, where the sulphate of quinine proved successful in arresting a violent and obstinate cephalalgia, after almost every other medicine had been tried in vain. Among other things the carbonate of iron was fully exhibited under the care of Mr. Hutchinson. The patient was a butler in a family, 41 years of age, who became affected, for the first time, with head-ache in October 1822. It was in June, 1823, that he came regularly under Mr. B's care. His symptoms then were, excruciating pain in the head, coming on in the evening, continuing during the night, preventing sleep entirely, and gradually receding towards morning. Sometimes, however, the pain would continue for days together without remission. The pain was accompanied by occasional acute stabs, or darts, from one side of the head to the other, striking down the upper part of the spine, and in the direction of the inferior maxillary nerve, bearing some affinity to tic douloureux. During the severity of the paroxysms his eyes were suffused with tears, and ferretty, his face flushed, the scalp tender to the touch. In the intervals, his face was pale, pulse below 80, small and feeble, tongue foul, appetite bad, bowels irregular, motions dark and offensive.

Mercurial alteratives, purgatives, bitter tonics, gave temporary relief only. Then were tried, cupping, blisters, purgation, but in vain. Tonic aperients and colchicum appeared to remove the disease, but it soon returned as bad as ever. Cinchona, valerian, shared the same fate, and the carbonate of iron seemed to aggravate the complaint. He went to Sandgate, where Mr. Hutchinson (brother of Mr. H. of Southwell) administered the carbonate of iron extensively, after bleeding. But this ultimately failed. Mr. Bushell now administered the sulphate of quinine in doses of one grain and a half every three hours. The dose was augmented. In four days the head-ach was gone; but the countenance was flushed, and the pulse very hard. The medicine was lowered. In ten days the patient was quite well, and has continued so.

We have had occasion lately to exhibit this medicine in some severe cases of hemicrania, with entire success. We shall only glance at one or two cases. A gentleman, attended by Mr. Bampfield, laboured under violent periodical pain in the right eye, and that part of the forehead, which rendered him almost distracted during the paroxysms, which came on once and sometimes twice a day. He was leeched, purged, &c. but still the pain recurred without mitigation. We then determined to give the sulphate of quinine, and also Fowler's solution of arsenic. These put a stop to the disease in a few days, and no return took place. In the case of a lady at Kensington we had similar success. She, for several years past, has had annual attacks of hemicrania of the right side, attended with redness and lacrymation of that eye, which, in some attacks continued several weeks, especially when it was treated by purgatives, on the supposition of its being of gastric or hepatic origin. In the present attack which was very violent, a smart purgative was first administered, and then a grain and a half of the sulphate ordered every six hours. Five drops of Fowler's solution was also taken thrice a day. In three days the paroxysms were rendered quite insignificant, and by the fifth day they ceased altogether, and did not return. Some instances have come to our knowledge, where drachm doses of the powder of valerian every four or six hours put a speedy termination to periodical hemicrania, after bark, arsenic, and many other medicines had entirely failed.

---

8. *Pulvis Antimonialis.* Dr. Hawkins, of Hitchin, made experiments with the pulvis antimonialis of the shops (some of which was procured from Apothecaries' Hall) carrying the dose as high as one drachm, without ever producing the slightest sensible effect on the constitution, or any function of it. He consequently comes to the conclusion that the medicine, on which we often place much dependence, is totally inert. We can hardly come to this conclusion; at the same time we are much inclined to fear that there is some foundation for the scepticism of Dr. Hawkins. The pulvis antimonialis is so generally given in combination with other medicines—and when given alone, is so seldom productive of effect, that we do not much wonder at the delusion under which we have laboured as to its medicinal properties. The tartrite of antimony should, we think, be trusted to instead of the antimonial powder.—*Ed. Journal, No. 2, New Series.*

---

9. *Cubebs in Gonorrhœa.* Dr. Crane, of Boston, Linconshire, has published some observations on the treatment of gonorrhœa by cubebs, in the last number of our Northern cotemporary. He observes, what is very true, that in most instances, the urine smells strongly after a few doses of the cubebs are taken—the discharge decreases—and the heat of urine is much relieved. It rather opens than confines the bowels—if otherwise, a few grains of blue pill, at bed time, will be proper, with a saline aperient in the morning. This medicine generally improves the appetite and exhilarates the spirits. In two cases, it produced irritation and mental anxiety. Small doses, he thinks, are of no service: the powder must be taken "in doses of from two to three drachms each, three or four times a day." In this we cannot agree with our author. Within the last month we have completely cured two cases of smart gonorrhœa by drachm doses thrice a day. In fact, the powder is so light and bulky that two or three drachms are not easily got down. Cold water is as good a vehicle as any. When the gonorrhœa is removed, the cubebs should be continued for ten days or a fortnight, gradually diminishing the dose. Sometimes a thin bluish coloured gleet, in small quantity, remains and cannot be removed by a continuance of the cubebs. In these cases, a weak solution of the sulphate of zinc injected twice or thrice a day, will prove efficient. Our author remarks, that if no good effect be produced by the cubebs in eight or ten days, it will be useless to continue the remedy. A combination of balsam copaiba is an exceedingly nauseous medicine, in Dr. Crane's opinion. We have found it useful, on several occasions, to exhibit the balsam as well as the cubebs in gonorrhœa, which is one of the most troublesome complaints which the medical practitioner has to deal with. The balsam is best borne at night going to bed in the dose of a good sized tea-spoonful. The cubebs may be taken through the day. In all cases, we find low living and quietude hasten the cure.

*Cubebs in Chronic Rheumatism.* Accident led Dr. Crane to employ cubebs in this almost incurable complaint. In a few instances it proved beneficial—in others, it gave no relief. It must be continued for some time to produce effect.

---

10. *Ammonia in Drunkenness.* We know not whether the discovery of a medicine, which could dissipate the effects of spirituous or vinous potations, would be desirable, in a general point of view. That it might save the lives of some, and the characters of many, annually, there can be no doubt—but that the knowledge of a specific of this kind would lessen the terrors and consequently increase the practice of inebriety, we are also confident. Within the last few years the liquor ammoniæ has been reported by some continental physicians as possessing the power of quickly bringing an intoxicated man to his senses. Further experience, however, has not tended to confirm this statement. Mr. Chevalier, of Paris, has recently published some cases shewing the total inefficacy of the ammonia, while a few others rendered it probable that the medicine had considerable effect in dissipating the vinous hallucination. What the circumstances are, which render the ammonia inert in one case and efficient in another, have not yet been ascertained. Sixteen or twenty drops of the liquor ammoniæ have been given as a dose, diluted in a wine glassful of water, and repeated pro re nata.

---

11. *Treatment of Typhus Fever.* In the March number of our respected cotemporary, the Medical Repository, Messrs. Parkinson have recommended a plan of treatment in typhous fevers, somewhat different from any that have lately been proposed.* It bears the nearest analogy to the *gestation in the open air*, mentioned by Celsus, and put in practice by Dr. Jackson. Messrs. Parkinson are surgeons to a pauper institution, and their plan is as follows:—"immediately on application for relief the patient is conveyed to the fever-ward. The body is then cleansed; and, if required, the warm bath is employed, the hair either thinned or entirely removed, and clean body linen, with an upper clothing of woollen, put on. Bleeding, if required, is then employed; and an emetic or cathartic, if hitherto omitted, and now required, is had recourse to, the cathartic medicine being repeated as circumstances may require. A blister is applied to the nape of the neck, and in the neighbourhood of any part in which congestion is threatened. The patient is then seated in a chair with a railed back, so high as well to support the head whilst leaning backwards; the legs, if required, being supported horizontally at a convenient height, and he is then placed in the current of fresh air passing upwards from the windows and the lower parts of the building through the openings in the ceilings and roof. As the patients gain strength, they are allowed to change their situations, either towards or from the windows; and, when able, which, in general, is within two or three days, they are permitted to walk about the ward; but are very rarely allowed to lay on the bed in the day-time.

"The regimen is composed of tea, toast-water, barley-water, gruel, milk-pottage, and, as the tongue becomes clean, broth is added; but

* It appears to have been recommended by the late Dr. Lettsom, in one of the volumes of the Medical Society's Memoirs.

beer, wine, &c. are prohibited through the progress of the disease, and even of recovery.

"The beds and pillows, with a view to prevent the accumulation of heat about the head and trunk, are loosely filled with straw. The patients are requested to lay as much as they are able on their sides, and are required to quit their beds by nine o'clock of the morning. The advantages derivable from this arrangement, as well as from the exclusion of the patients from their beds during the day-time, will be perceived, on ascertaining, by interposing the hand between the back of the patient and the subjacent bed-clothes, the vast degree of heat which is there accumulated.

"The results from this mode of treatment have been extremely satisfactory. Direct amendment and speedy recovery have been obtained in a very large proportion of cases. Out of 187 patients, only five were lost: two of whom were between seventy and eighty years of age; two others were reduced to a hopeless state of weakness previous to their application; and one sunk from extensive sphacelations, induced by previous long-continued confinement to her bed.

"Such success excited the hope that, by avoiding stimulants, opening the bowels, keeping the patients in an *erect position, and allowing the free access of cold air*, the destructive powers of this malady might be effectually opposed. A longer trial has not only confirmed this hope, but made it appear to be a duty to make known the results in those cases in which it has been employed" 199.

Our authors observe, "that corresponding success is very seldom obtained when the attempt is made in the chambers of those who are surrounded by the luxuries or even the comforts of life." We object not to the cooling mode of treatment recommended above; but we confess we cannot see on what principle the utility of the upright posture can be explained. Coolness enough might be obtained without this; and whoever has laboured under fever must remember what distressing sensations and increased velocity of pulse are occasioned by the perpendicular posture. We bow, however, to the authority of *facts*, if experience shall prove them such in the hands of the many.

---

12. *Stibiated Mercurial Ointment.* An Italian physician, (Dr. Miccoli,) avers that, for *forty years* past, he has had the greatest success in practice, from using an ointment composed of one ounce of quicksilver, two drachms of lard or suet, and five scruples of antimonial powder. In the first place, he discovered that frictions with this ointment *never salivated*—a statement to which we are not disposed to give implicit credence—and in the second place, encouraged by this discovery, he used the ointment in dartrous affections, rheumatism, gout, obstructions, old ulcers, *phthisis*, ophthalmia, *impotence*, periodical diseases, congestions, cough, dropsy, asthma, tumours, &c. with the most happy effects. (les plus heureux effets.) It is difficult not to smile at such statements:—yet they are put forth gravely, and copied by many of our continental cotemporaries. The ointment was rubbed on the thighs, groins, and armpits. He affirms that, in many instances, he prevented hydrophobia by these frictions ! ! Dr. Miccoli avers further, that crude mercury, triturated with antimonial powder, (poudre de Pearson) and administered in doses of from nine to twelve grains twice or thrice a day, produced the same effect as the pommade divine abovementioned.

Without putting much faith in the therapeutical statements of our author, we would be glad to know whether antimony in the above form has the power ascribed to it of preventing the mercurial influence on the system, as manifested by ptyalism. We know that combinations of mercury and antimony, *in other forms*, have the power of inducing salivation readily enough.

---

13. *Contra Ptyalismum.* We all know that hitherto nothing has been found effectual in reducing the action of mercury on the salivary glands, when ptyalism has once taken place, but time. Neither do we think that a constitutional afflux of this kind could be checked, by external means at least, with safety to the patient. Solutions of the acetate of lead have been recommended as gargles. More recently Dr. Kreugerhausen, of Gostrow, affirms that he has, in many cases, quickly suppressed mercurial ptyalism by tar, taken into the mouth and diffused over the tongue and neighbouring parts. The remedy is not the most agreeable in taste ; but we think it might be made more palatable in the form of gargles, should any practitioner be inclined to give it a trial.

---

14. *Hospital Reports.* We are glad to see the medical officers of public institutions beginning to publish their Hospital Reports more generally than formerly. They would thus preserve them from those harpies who occasionally give to the public distorted representations of cases.

Dr. Chisholm has followed the example of his colleague Dr. Carter, in giving accounts, from time to time, of the prominent cases that come before him in the Kent and Canterbury Hospital. His first report is published in our respected cotemporary, the Medical Repository for March, and contains much interesting matter, which is, however, incapable of analysis. We can only glance at one or two subjects.

I. *Melena.* A man was admitted into the hospital, on the 27th June 1823, with pain and sense of fulness in both hypochondria—debility— stools frequent, pitchy, and generally mixed with dark grumous blood. Six drachms of oil of turpentine with half an ounce of oil of almonds were exhibited ; while decoction and tincture of cinchona were administered through the day. Under this treatment he greatly improved, and was discharged cured. Our author observes, that he has been in the habit for many years past of giving turpentine in cases of melena (and some others) often in much larger doses than in this case, and he has found it a most valuable medicine.

II. *Colchicum in Case of Tape Worm.* A respectable farmer had been afflicted with tape worm for ten years, and had tried, under various practitioners, the whole round of anthelmintics, with only temporary benefit. His farrier too, "had given him turpentine enough to kill a horse," but not sufficient to kill the worm. Having met with an instance where vinum colchici given for rheumatism expelled a quantity of tape-worm, Dr. Chisholm was induced to give it a trial in the present case. He therefore furnished the patient with two ounces of the wine (made according to Dr. Marcet's formula) desiring him to take a tea-spoonful in a little water two or three times a day.* On the third or fourth day he passed a large quantity of the worm, and he continued to take the medicine for a week, but did not pass any more worm. He has now been free from the tape-worm more than three years. He took no medicine in the last instance but the colchicum.

---

15. *Lemon Juice in Scurvy.* It has long been known to many intelligent observers that salt provisions are not the only cause of scurvy, and that lemon juice is by no means an infallible cure for the disease, however induced, notwithstanding the evidence of Sir Gilbert Blane so positively advanced to the contrary. In support of our position we shall here bring forward an abstract from an official document of unquestioned authenticity and recent occurrence.

In the year 1822, His Majesty's ship Leander, sailed from Trincomalee for the Cape of Good Hope, taking on board the mechanics of the Dock Yard establishment then reduced on the island. There were also embarked twenty-six invalids and all the sick that could be removed from the hospital. These invalids and sick were principally affected with chronic hepatitis, dysentery, and phthisis pulmonalis, all of which (even some who were expectorating large quantities of purulent matter) recovered on the passage to the Cape. This good fortune was counterbalanced by scurvy, which broke out among the crew, and, in spite of large quantities of lemon juice plentifully administered, in conjunction with every other antiscorbutic which the ship could produce, spread to an alarming extent, and in one case proved fatal. Had they not reached the Cape at the time they did, the Leander would have presented as deplorable a spectacle as the Anson, at Juan Fernandez, notwithstanding the supposed infallible specific, *lemon juice*, which, in no instance, on board the Leander, had the slightest effect in even checking the ravages of scurvy. Immediately the ship reached the Cape, and the crew got plenty of fresh *animal food*, in conjunction with vegetables, they rapidly recovered.† Specimens of the lemon juice used were transmitted to the Victualling Board, and carefully analyzed in London. It was found to be perfectly good.

---

* This dose is rather large. We would not advise such a quantity to be commonly prescribed. In the present instance there was probably less danger, as the farmer's bowels appear to have been pretty strong.

† See Mr. Bampfield's remarks also on this subject, in his valuable work on tropical dysentery.

